# Transcriptional Analysis of Cotton Bollworm Strains with Different Genetic Mechanisms of Resistance and Their Response to *Bacillus thuringiensis* Cry1Ac Toxin

**DOI:** 10.3390/toxins14060366

**Published:** 2022-05-25

**Authors:** Shan Yu, Chenyang Wang, Kaixia Li, Yihua Yang, Ya-Zhou He, Yidong Wu

**Affiliations:** 1 Key Laboratory of Integrated Pest Management on Crops in East China (MARA), College of Plant Protection, Nanjing Agricultural University, Nanjing 210095, China; 20210108@jaas.ac.cn (S.Y.); cywang@stu.njau.edu.cn (C.W.); 2018202047@njau.edu.cn (K.L.); yhyang@njau.edu.cn (Y.Y.); 2 Institute of Plant Protection, Jiangsu Academy of Agricultural Sciences, Nanjing 210014, China

**Keywords:** *Helicoverpa armigera*, Cry1Ac toxin, insect resistance, cadherin, ABC transporter, tetraspanin, Cry1Ac response

## Abstract

Transgenic crops producing *Bacillus thuringiensis* (Bt) insecticidal proteins are grown widely for pest control, but the evolution of resistance in target pests could reduce their efficacy. Mutations in genes encoding cadherin, ABC transporter or tetraspanin were linked with resistance to Cry1Ac in several lepidopteran insects, including the cotton bollworm (*Helicoverpa armigera*), a worldwide agricultural pest. However, the detailed molecular mechanisms by which these mutations confer insect resistance to Cry1Ac remain largely unknown. In this study, we analyzed the midgut transcriptomes of a susceptible SCD strain and three SCD-derived Cry1Ac-resistant strains of *H. armigera* (SCD-r1, with a naturally occurring deletion mutation of *cadherin*; SCD-KI, with a knock-in T92C point mutation in *tetraspanin*; and C2/3-KO, with both *ABCC2* and *ABCC3* knocked out). Evaluation of midgut transcript profiles of the four strains without Cry1Ac exposure identified many constitutively differentially expressed genes (DEGs) in the resistant SCD-r1 (n = 1355), SCD-KI (n = 1254) and C2/3-KO (n = 2055) strains. Analysis of DEGs in the midguts of each strain after Cry1Ac exposure revealed similar patterns of response to Cry1Ac in the SCD and SCD-r1 strains, but unique responses in the SCD-KI and C2/3-KO strains. Expression of midgut epithelium healing and defense-related genes was strongly induced by Cry1Ac intoxication in the SCD and SCD-r1 strains, while immune-related pattern recognition receptor and effector genes were highly expressed in the SCD-KI strain after Cry1Ac exposure. This study advances our knowledge of the transcriptomic basis for insect resistance to Bt toxins and provides a valuable resource for further molecular characterization of insect response to Cry1Ac toxin in *H. armigera* and other pest species.

## 1. Introduction

Genetically engineered crops that produce insecticidal proteins from the bacterium *Bacillus thuringiensis* (Bt) have revolutionized pest control [1,2]. These Bt proteins kill some devastating insect pests, but cause little or no harm to most other non-target organisms, including humans [3,4,5]. Worldwide planting of Bt crops increased from 1.1 million hectares in 1996 to 190.4 million hectares in 2019 [6]. Benefits of Bt crops include pest suppression, reduced insecticide usage and increased yields and farmer profits [7,8,9,10,11]. However, these benefits of Bt crops have been diminished by the evolution of pest resistance. To date, field-evolved resistance that has practical consequences for pest control has been observed in populations of at least ten major pest species [12,13,14], which greatly threatens the continued success of Bt crops. A detailed knowledge of the molecular basis of insect resistance to Bt toxins is of great importance for effective pest resistance management.

The most widely used Bt toxins are the 3-domain Cry toxins, for example, Cry1Ac in transgenic cotton that kills certain Lepidopteran larvae [2]. Upon ingestion by an insect, the Cry protoxins are solubilized in the gut and cleaved by gut digestive proteases to yield activated toxins that bind to specific membrane-associated receptors, such as cadherin and ATP-binding cassette (ABC) transporter family proteins [2,13,15]. The interactions of Cry toxins with their midgut receptors trigger toxin oligomerization and pore formation, resulting in osmotic imbalance and enterocyte death promoted by compensatory water influx through aquaporins [16]. The most common mechanism of Bt resistance in insects is via disruption of toxin binding to these midgut receptors. Resistance to Cry toxins is associated with mutations or expression alterations of cadherins and ABC transporters in at least four and eight lepidopteran insects, respectively [13,17,18,19,20,21]. In addition to these well-established receptor genes, a point mutation (T92C) in a tetraspanin gene (*HaTSPAN1*) confers dominant resistance to Cry1Ac in a field strain of cotton bollworm, *Helicoverpa armigera* [22]. Although the causality between alterations of these genes and the development of resistance to Cry toxins in insects is clear, the detailed molecular mechanisms underlying this process remain largely unknown.

The cotton bollworm is one of the most destructive insect pests of cotton, corn, vegetables and other crops worldwide [23]. In China, Bt cotton expressing Cry1Ac was employed for the control of this major pest since 1997. Long-term exposure of field populations to Bt cotton has caused increases in the frequency of resistance to Cry1Ac in cotton bollworms [24,25]. A disrupted allele (*r1*) of the cadherin gene carrying a deletion between exons 8 and 25 that introduces a premature stop codon is genetically linked with resistance to Cry1Ac in the GYBT strain of *H. armigera* [26]. Introgression of the *r1* allele into a susceptible SCD strain led to 438-fold resistance to Cry1Ac in the introgressed SCD-r1 strain [27]. In addition, CRISPR/Cas9-mediated introduction of the T92C mutation in *HaTSPAN1* into the SCD strain conferred 125-fold resistance to Cry1Ac in the knock-in strain SCD-KI [22]. Furthermore, simultaneous knockout of the two ABC transporter genes *HaABCC2* and *HaABCC3* by CRISPR/Cas9 caused >15,000-fold resistance to Cry1Ac in the SCD knock-out strain C2/3-KO [20]. Therefore, mutations of genes within at least three distinct families can give rise to Cry1Ac resistance in *H. armigera*. However, whether different genetic mutations lead to Cry1Ac resistance through the same or different molecular mechanisms remains poorly understood. Here, we analyzed the transcriptional responses of the susceptible SCD and the three SCD-derived resistant cotton bollworm strains to Cry1Ac exposure using RNA-sequencing (RNA-seq). We found that the three resistant strains exhibited distinct patterns of response to Cry1Ac exposure, indicating that each of the three mutations confers resistance to Cry1Ac via a different molecular mechanism. Our study gives a better understanding of the transcriptomic basis for insect resistance to Bt toxins and provides valuable information for further study on molecular mechanisms of the interactions between insects and Bt toxins.

## 2. Results

### 2.1. Overview of the Midgut Transcriptomes of Four Cotton Bollworm Strains with or without Cry1Ac Exposure

We used RNA-seq to investigate the transcriptional responses of cotton bollworm strains with different genetic mechanisms of resistance to Cry1Ac exposure. Early fifth instar larvae from the susceptible SCD strain and the three resistant strains (SCD-r1, SCD-KI, and C2/3-KO) were fed with Cry1Ac (10 μg per insect) or solvent (PBS, negative control), and the midguts were dissected at 6 h after the feeding and used for RNA-seq. RNA libraries were constructed with three biological replicates for each treatment and sequenced to the total depth of 1.02 billion reads, 75% of which were mapped to the *H. armigera* genome (Table S1). Read counts for 15,656 genes were normalized to numbers of genome-mapped reads in each library, expressed as fragments per kilobase per million (FPKM) (Table S2). Correlation analysis of gene expression between samples showed that all three biological replicates correlated well in each treatment, confirming the repeatability of our RNA-seq data (Figure S1).

### 2.2. Effects of Different Genetic Mutations on Midgut Transcriptomes of Cotton Bollworms without Cry1Ac Exposure

**Figure 1:**
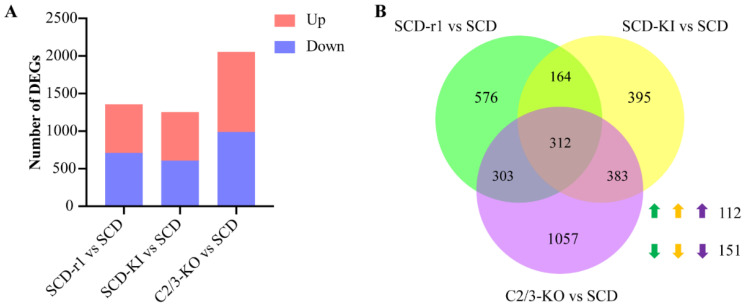
Analysis of differentially expressed genes (DEGs) between resistant and susceptible strains without Cry1Ac exposure. (**A**) Number of DEGs in three resistance strains when compared with the susceptible SCD strain. (**B**) Venn diagram analysis of DEGs in the three resistance strains. Up-arrows indicate co-upregulated genes and down-arrows indicate co-downregulated genes.

Before analyzing the midgut transcriptional responses to Cry1Ac exposure, we first investigated whether the mutations in these resistance-related genes would have an impact on the midgut transcriptome of cotton bollworms without Cry1Ac exposure. Strikingly, we found 645 upregulated and 710 downregulated genes in the midguts of the SCD-r1 strain, 645 upregulated and 609 downregulated genes in the midguts of the SCD-KI strain, and 1066 upregulated and 989 downregulated genes in the midguts of the C2/3-KO strain, compared to the SCD strain (Figure 1A and Tables S3–S5). Among these differentially expressed genes (DEGs), 312 were shared between the three datasets, with 112 were upregulated and 151 were downregulated in all three resistant strains (Figure 1B and Table S6).

To analyze the function of these DEGs in the three resistant strains, we first performed gene ontology (GO) analysis. The most significantly enriched 10 (Top 10) GO terms were shown in Figure S2. The DEGs in all three resistant strains showed significant enrichment in metabolic process, catalytic activity, organic substance metabolic process and DNA integration (Figure S2 and Tables S3–S5), that may represent the general impact of different mutations on the *H. armigera* larvae midgut physiology. In addition, some of the DEGs in each strain exhibited specific GO enrichment. For example, 4 out of the top 10 GO terms (innate immune response, aminoglycan catabolic process, carbohydrate metabolic process, and monooxygenase activity) were only enriched by the DEGs in the C2/3-KO strain (Figure S2 and Table S5).

**Figure 2:**
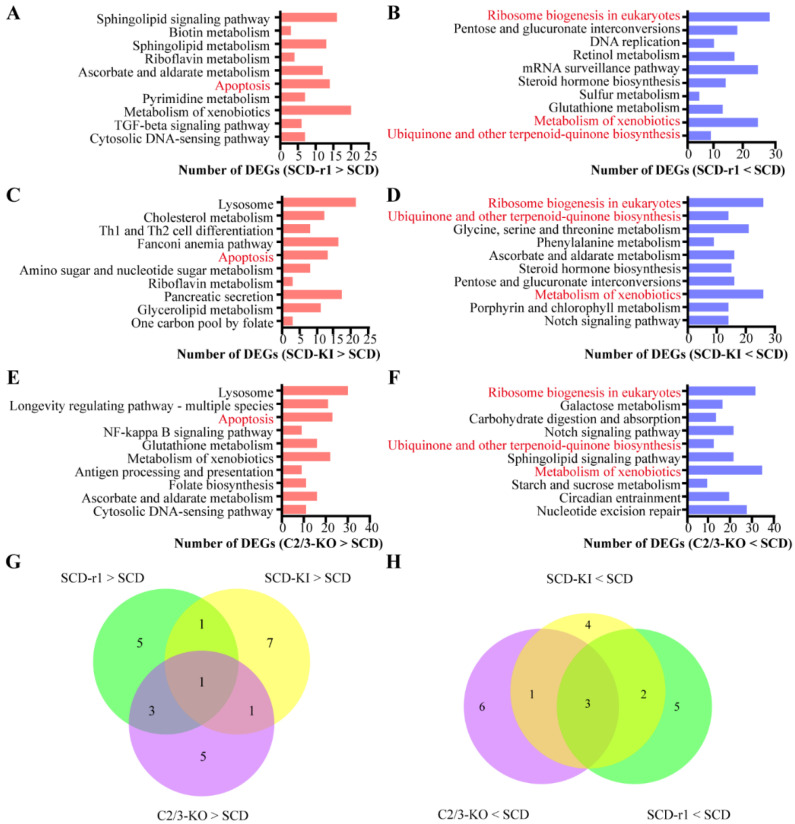
Functional analysis of DEGs between the three resistance strains and the susceptible SCD strain without Cry1Ac exposure. (**A**–**F**) The most significantly (*p* < 0.05) enriched 10 (Top 10) pathways for the constitutively upregulated (**A**,**C**,**E**) or downregulated (**B**,**D**,**F**) genes in the three resistant strains. The column indicates the number of DEGs related to each pathway. (**G**,**H**) Venn diagram analysis of the top 10 pathways enriched by constitutively upregulated (**G**) or downregulated (**H**) genes in the three resistance strains.

We then performed KEGG pathway analysis for the upregulated and downregulated genes in each strain, respectively, to further characterize the function of those DEGs. The results indicated that the DEGs associated with each resistant strain enriched in distinct functional groups. The Top 10 KEGG pathways for the up or downregulated genes in each strain are shown in Figure 2. For the SCD-r1 strain, some upregulated genes were significantly enriched in sphingolipid signaling pathway, sphingolipid metabolism and apoptosis. For the SCD-KI strain, some upregulated were involved in lysosome, cholesterol metabolism and apoptosis. Many upregulated genes in the C2/C3-KO strain exhibited significant enrichment in lysosome, longevity regulating pathway and apoptosis. Of the upregulated genes enriched Top 10 KEGG pathways, only apoptosis was shared between the three resistant strains (Figure 2A,C,E,G). In addition, only three terms (ribosome biogenesis, metabolism of xenobiotics, ubiquinone and other terpenoid–quinone biogenesis) were shared among the Top 10 KEGG pathways enriched for downregulated genes in the three resistant strains (Figure 2B,D,F,H). Together, these data indicate that each of the three resistance-conferring mutations has a significant yet unique impact on gene expression in the midguts of cotton bollworms without Cry1Ac exposure.

### 2.3. Effects of Cry1Ac on Midgut Transcriptomes of Susceptible and Resistant Cotton Bollworms

**Figure 3:**
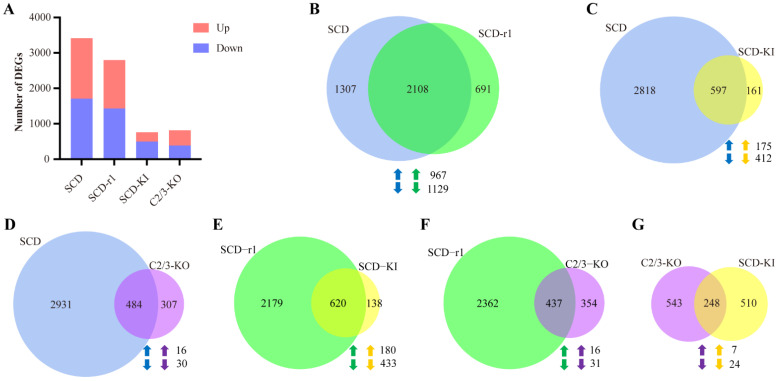
Analysis of DEGs in Cry1Ac-treated larvae as compared to the control larvae for each strain. (**A**) Number of DEGs in Cry1Ac-treated larvae when compared to the control larvae for each strain. (**B**–**G**) Venn diagram analysis of the DEGs in paired strains. Up-arrows indicate co-upregulated genes and down-arrows indicate co-downregulated genes.

We then assessed the transcript profiles of Cry1Ac-treated larvae as compared to the control larvae for each strain. We found 1707 upregulated and 1708 downregulated genes in the SCD midgut, 1369 upregulated and 1430 downregulated genes in the SCD-r1 midgut, 263 upregulated and 495 downregulated genes in the SCD-KI midgut, and 433 upregulated and 358 downregulated genes in the C2/3-KO midgut after Cry1Ac exposure (Figure 3A and Tables S7–S10). Therefore, more genes were affected by Cry1Ac exposure in the SCD (3415) and SCD-r1 (2799) strains compared with the SCD-KI (758) and C2/3-KO (791) strains. GO analysis revealed enrichment of the DEGs from different strains in both the same and distinct functional groups (Figure S3 and Tables S7–S10). Of the top 10 GO terms, two (catalytic activity and oxidoreductase activity) were enriched by the DEGs in all four strains. However, specific enrichment of the DEGs from each strain was readily observed. For instance, 6 out of the Top 10 GO terms (hydrolase activity, UDP-glycosyltransferase activity, iron ion binding, oxidoreductase activity-acting on the CH-CH group of donors, transferase activity-transferring hexosyl groups, and peptidase activity-acting on L-amino acid peptides) were only enriched by the DEGs in the C2/3-KO strain (Figure S3). Further details of the GO terms enriched by the DEGs in each strain were shown in Figure S3 and Tables S7–S10.

**Figure 4:**
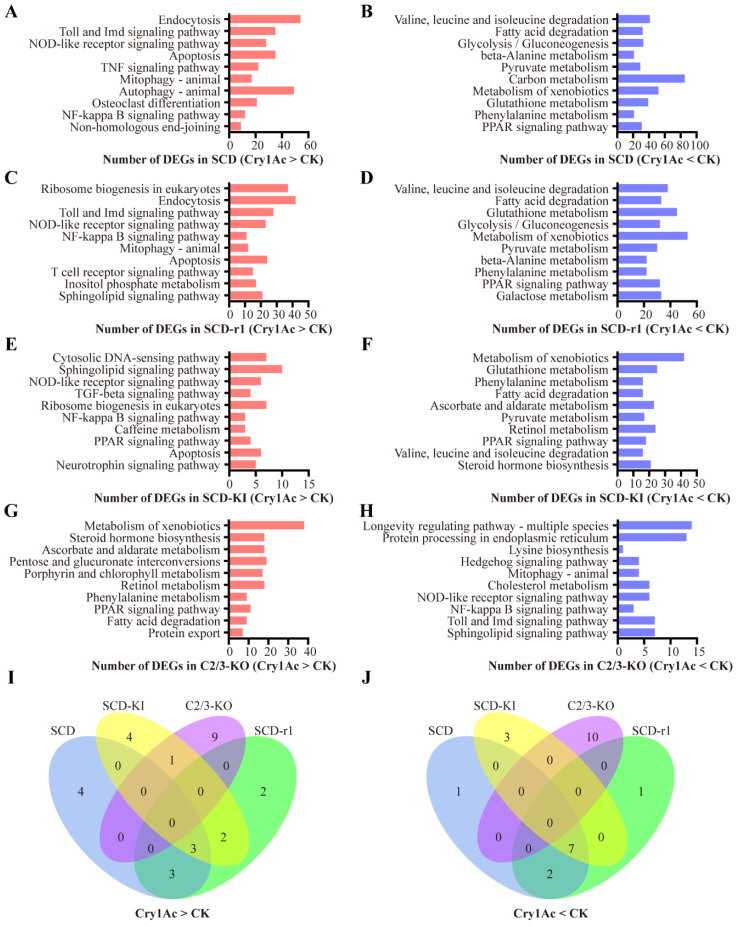
Functional analysis of DEGs in Cry1Ac-treated larvae as compared to the control larvae for each strain. (**A**–**H**) The top 10 pathways (*p* < 0.05) for the upregulated (**A**,**C**,**E**,**G**) or downregulated (**B**,**D**,**F**,**H**) genes in each strain in response to Cry1Ac exposure. The column indicates the number of DEGs related to each pathway. (**I**,**J**) Venn diagram analysis of the top 10 pathways enriched by upregulated (**I**) or downregulated (**J**) genes in the four strains after Cry1Ac exposure.

Notably, 2108 DEGs (account for 62% DEGs in the SCD strain and 75% DEGs in the SCD-r1 strain) were shared between the SCD and SCD-r1 strains, with 967 were upregulated and 1129 were downregulated in both strains, suggesting similar patterns of response to Cry1Ac within these two strains (Figure 3B). Functional analysis of the DEGs in the two strains further supported this hypothesis, as 6 of the Top 10 KEGG pathways enriched for the upregulated genes were shared between the SCD and SCD-r1 strains, including endocytosis, Toll and IMD signaling pathway, NOD-like receptor signaling pathway, apoptosis, mitophagy and NF-kappa B signaling pathway. Moreover, the downregulated genes in these two strains were enriched in almost the same KEGG pathways, of which many are related to metabolism processes (Figure 4A–D,I,J and Tables S7 and S8). Unlike the SCD-r1 strain, the response of the SCD-KI or C2/3-KO strain to Cry1Ac was quite different from that of the SCD strain both in terms of the number of co-regulated genes and the function of DEGs (Figure 3C,D and Figure 4).

Analysis of DEGs from the three resistant strains after Cry1Ac exposure relative to the control revealed unique responses of cotton bollworms with different genetic mechanisms of resistance to Cry1Ac. Of the 2799 DEGs in the SCD-r1 strain, only 22% and 16% were shared by the SCD-KI and C2/3-KO strains, respectively (Figure 3E,F). Moreover, merely 7 upregulated and 24 downregulated genes were common between the SCD-KI and C2/3-KO strains (Figure 3G). In addition, of the top 10 DEGs-enriched KEGG pathways, none were shared among the three resistant strains (Figure 4I,J). The C2/3-KO strain exhibited a strikingly different response to Cry1Ac exposure relative to the other strains, as 9 of the Top 10 KEGG pathways enriched for upregulated genes and all the Top 10 KEGG pathways enriched for downregulated genes were unique to the C2/3-KO strain (Figure 4I,J and Table S10).

### 2.4. Expression Profiles of Midgut Epithelium Healing Genes

**Figure 5:**
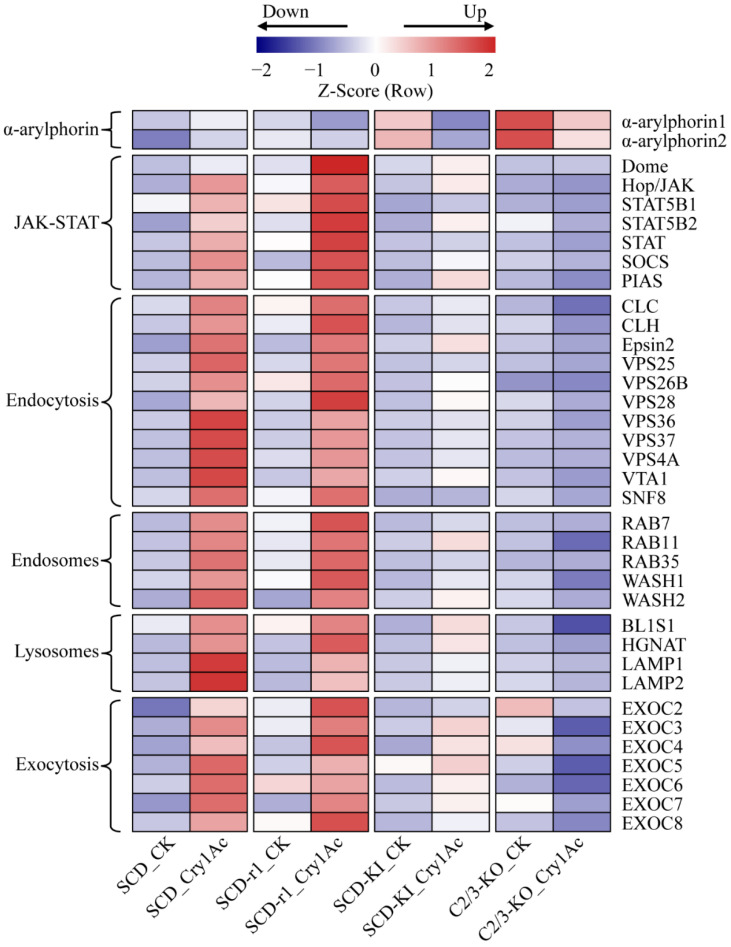
Expression profiles of genes related to midgut epithelium healing in the four cotton bollworm strains with or without Cry1Ac exposure. Expression levels are represented by the average of normalized expression from three biological replicates.

During Bt toxin intoxication, the insect midgut epithelium is damaged due to enterocyte death induced by osmotic imbalance [13]. However, insects can restore epithelium integrity by midgut cell regeneration and by repairing the injured membranes [28,29]. Therefore, the insect ability to regenerate disrupted gut cells contributes to the insect resistance to Bt toxins. We thus analyzed the expression profiles of midgut epithelium healing genes in the midguts of the four cotton bollworm strains with or without Cry1Ac exposure. It has been reported that α-arylphorin is an important factor promoting insect midgut stem cell proliferation after exposure to Bt toxins [30,31,32]. The transcript levels of two α-arylphorin genes in the larva midguts of the three resistant strains were higher than that of the susceptible SCD strain without Cry1Ac exposure, suggesting constitutive upregulation of α-arylphorins in resistant strains of *H. armigera* (Figure 5 and Table S11). However, while α-arylphorin expression was increased in the SCD strain, it was reduced in the three resistant strains by Cry1Ac intoxication (Figure 5 and Table S11). Therefore, whether the constitutive upregulation of α-arylphorins in the three resistant strains contributes to their enhanced tolerance to Cry1Ac warrants further investigation.

The janus kinase/signal transducer and activator of transcription (JAK/STAT) pathway functions in the regulation of insect midgut epithelium renewal via stem cell proliferation and differentiation after injury [33,34]. The expression levels of several key components (Dome, JAK and STAT) of this signaling pathway were strongly upregulated in the midguts of Cry1Ac-treated SCD and SCD-r1 larvae when compared to the non-treated control. A relative weaker upregulation of these genes by Cry1Ac exposure was also observed in the SCD-KI strain, but not in the C2/3-KO strain (Figure 5 and Table S11). Surprisingly, the expression of two negative regulators of JAK/STAT pathway, suppressor of cytokine signaling (SOCS) and protein inhibitor of activated STAT (PIAS) [35], also increased in the midguts of the SCD, SCD-r1 and SCD-KI larvae after Cry1Ac treatment (Figure 5 and Table S11). These results indicate that the JAK/STAT pathway was well coordinated in Cry1Ac-treated cotton bollworms and may play an important role in regulating midgut homeostasis after exposure to Cry1Ac.

The vesicle trafficking pathways are involved in promoting host plasma membrane repair after bacteria pore-forming toxins exposure [36]. The cell-surface-associated toxins can be internalized into cells through endocytosis, transported by endosome systems and finally degraded within lysosomes or recycled to the cell membrane via exocytic events [37]. Many genes related to these vesicle-trafficking pathways were upregulated in the midguts of the SCD, SCD-r, and SCD-KI larvae after Cry1Ac exposure relative to the control (Figure 5 and Table S11). The Cry1Ac-induced expression of these genes was generally higher in the SCD and SCD-r1 strains when compared with the SCD-KI strain. Conversely, Cry1Ac exposure reduced the expression of most of these genes in the C2/3-KO strain (Figure 5 and Table S11). These data suggest that the vesicle trafficking pathways were activated by Cry1Ac exposure in insects susceptible or with lower levels of resistance (SCD-r1 and SCD-KI) to Cry1Ac and may be involved in restoring gut epithelium integrity after Cry1Ac intoxication.

### 2.5. Expression Profiles of Defense Related Genes

**Figure 6:**
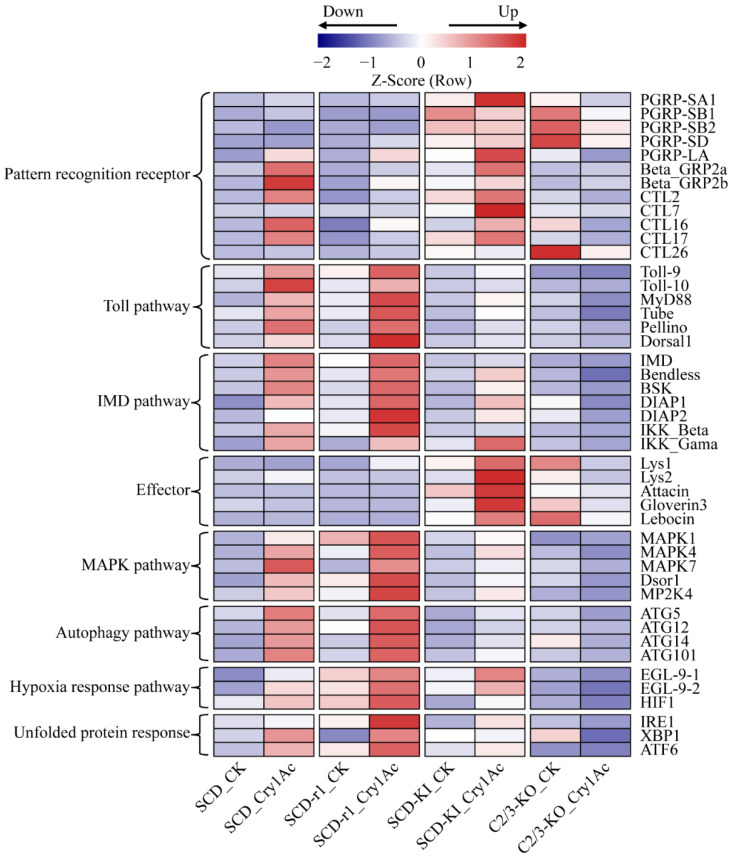
Expression profiles of defense related genes in the four cotton bollworm strains with or without Cry1Ac exposure. Expression levels are represented by the average of normalized expression from three biological replicates.

Studies have shown that insect innate immune responses are involved in host defense against Bt and/or its toxins [38,39]. We then investigated whether altered immune system is associated with resistance to Cry1Ac in cotton bollworms. The Toll and IMD signaling pathways play a central role in insect immunity against bacterial infection [40,41]. These pathways are activated by the binding of pattern recognition receptors (PRRs) such as peptidoglycan recognition protein (PGRP), beta-1,3-glucan recognition protein (beta-GRP), and C-type lectin (CTL) to pathogen-associated molecular patterns on the surface of invading microorganisms, finally leading to the synthesis of various immune effectors [42]. Compared with the control group, the expression of several genes encoding PRRs and key components of the Toll and IMD pathways increased in the midguts of Cry1Ac-treated SCD larvae, suggesting activation of these immune pathways by Cry1Ac intoxication in susceptible insects. Cry1Ac exposure also upregulated the expression levels of these immune-related genes in the SCD-r1 and SCD-KI strains, but not in the C2/3-KO strain (Figure 6 and Table S11). Notably, compared with the SCD and SCD-r1 strains, the transcript levels of several PRR and effector genes were higher in the midguts of the SCD-KI and C2/3-KO strains without Cry1Ac exposure, indicating constitutive overexpression of these genes in the SCD-KI and C2/3-KO strains (Figure 6 and Table S11).

Several intrinsic cellular defense pathways, such as the mitogen-activated protein kinase (MAPK) pathways, the autophagy pathway, the hypoxia response pathway, and the unfolded protein response, were reported to counter the toxic effects of Bt toxins in invertebrates [29]. The expression levels of a number of genes related to these intracellular signaling processes were upregulated in Cry1Ac-treated SCD, SCD-r1 and SCD-KI larvae compared to the corresponding non-treated control, suggesting activation of defense signaling pathways in these strains by Cry1Ac intoxication (Figure 6 and Table S11). The transcript levels of most of these genes were higher in the SCD-r1 larvae, but lower in the SCD-KI larvae, when compared with that in the SCD larvae after Cry1Ac treatment. In contrast, the expression of most of these genes was repressed by Cry1Ac exposure in the C2/3-KO strain (Figure 6 and Table S11). Taken together, these results indicate that the three cotton bollworm strains with different genetic mechanisms of resistance exhibited various defense responses to Cry1Ac exposure.

## 3. Discussion

Resistance alleles based on mutations to the cadherin, ABC transporter or tetraspanin genes are well known to affect the efficacy of Cry1Ac toxin [13,20,22,27,43]. However, how different genetic mutations confer the same type of insect resistance remains largely unexplored. In the current study, we analyzed the global midgut transcriptional profiles of a susceptible and three resistant strains of *H. armigera* with or without Cry1Ac exposure. Importantly, all the three resistant strains were derived from the susceptible SCD strain, and each of them carries a mutation in one of the above three resistance-related genes. Therefore, the four strains constitute a perfect system for deciphering the molecular mechanisms underlying insect resistance conferred by different genetic mutations. Compared with the susceptible SCD strain, many genes showed constitutively transcriptional differences in the resistant SCD-r1, SCD-KI, and C2/3-KO strains, demonstrating that all these mutations have significant impacts on the midgut gene expression in *H. armigera*. It seems that different mutation leads to different transcriptional changes in the cotton bollworm midgut. However, 112 upregulated and 151 downregulated genes were common in all three resistant strains used in our study (Table S6). The differential expression of these genes may reflect a general impact of different resistance-conferring mutations on cotton bollworm larvae and, thus, has the potential to be used as molecular indicators for the rapid detection of resistant insects in the field. We also found that the three resistant strains exhibited distinct patterns of response to Cry1Ac exposure, suggesting that each of the three mutations may confer resistance to Cry1Ac via a different molecular mechanism. Our data showed the molecular complexity of insect resistance to Bt toxins.

Cadherins belong to a superfamily of transmembrane proteins that are involved in a variety of biological processes, such as cell adhesion, cell migration, cytoskeletal organization, cell signaling and morphogenesis [44]. Here, we found that the loss-of-function mutation of the cadherin gene resulted in constitutively differential expression of 1355 genes in the midguts of the SCD-r1 strain compared with the susceptible SCD strain, including many genes related to metabolism of xenobiotics, sphingolipid signaling pathway, ribosome biogenesis and mRNA surveillance pathway. These results indicate an important role of the cadherin protein in maintaining a normal midgut function in *H. armigera*. In line with this hypothesis, a fitness cost is associated with the functional disruption of cadherin in *H. armigera*, as the larval development time of the SCD-r1 strain was significantly longer than that of the SCD strain [27]. Our data may help to explain the fitness cost linked to the cadherin mutation in the SCD-r1 strain.

The relationship between cadherin disruption and Bt resistance was studied in detail. The first resistance-conferring mutation of cadherin was found in tobacco budworm *Heliothis virescens*, where the truncation of a cadherin gene caused by a retrotransposon was linked to resistance against Cry1Ac [18]. Subsequently, a variety of cadherin mutations were found in field Bt-resistant populations of *Pectinophora gossypiella*, *H. armigera* and *H. punctigera* [45,46,47]. Knockout of a *H. armigera* cadherin gene by CRISPR/Cas9 further confirmed the importance of cadherin disruption in Cry1Ac resistance [43]. Despite the significant role of cadherin mutation in the development of insect resistance to Cry1Ac, similar patterns of response to Cry1Ac exposure were obtained in the SCD-r1 and SCD strains both in terms of the number and function of DEGs in the midguts of Cry1Ac-treated larvae when compared with the non-treated control. This suggests that the mode of action of Cry1Ac within the SCD-r1 strain was not significantly changed. According to the pore insertion model, binding of Cry toxin monomers with cadherin accelerates the N-terminal α1-helix cutting off, making the toxin monomers could more efficiently form the “prepore” oligomer in solution [48]. However, some background cleavage of the α1-helix may occur because insects lacking a functional cadherin protein can also be killed by Cry toxins [15,49]. Therefore, the disruption of cadherin in SCD-r1 might reduce the efficiency of Cry1Ac oligomerization in the midguts. Nevertheless, the process was not blocked and the midgut epithelial cells of SCD-r1 larvae were also damaged and, thus, showed a similar response to that of the SCD larvae after Cry1Ac exposure.

Notably, many genes related to midgut epithelium healing and defense signaling pathways, such as the JAK/STAT, vesicle trafficking, and intrinsic cellular defense pathways, were strongly upregulated in the midguts of the SCD and SCD-r1 larvae in response to Cry1Ac exposure (Table S11), indicating the involvement of these signaling processes in the interactions of cotton bollworms with Cry1Ac. Increased stem cell proliferation rate was observed when *Achaea janata* larvae were exposed to sublethal concentrations of a Bt formulation. This higher proliferation rate was supposed to allow larvae to repair their gut epithelium [50]. The vesicle trafficking pathways protected *Caenorhabditis elegans* against Cry5B by eliminating bound Cry5B protein pores [51]. In addition, the MAPK, autophagy, hypoxia response and unfolded protein response pathways are utilized in *C. elegans* protection against Cry proteins [29]. Future work is needed to explore whether and how these Cry1Ac-responsive signaling pathways participate in insect tolerance/resistance against Bt toxins. Recently, it was reported that an activated MAPK cascade modulates the expression of several midgut genes related to Cry protein susceptibility and causes resistance to Cry1Ac in *Plutella xylostella* [52,53]. Therefore, activation of the MAPK pathways may represent a self-protective mechanism utilized by insects to reduce its susceptibility to subsequent Cry toxins following a prior exposure.

Generally, the Toll and IMD signaling pathways are activated by the binding of host PRRs (PGRP, beta-GRP and CTL) to pathogen-associated molecular patterns on the surface of invading microorganisms [40,41,42]. For example, recognition of bacteria is achieved through the sensing of specific forms of peptidoglycan, an essential glucopeptidic polymer restricted to the cell wall of both Gram-negative and Gram-positive bacteria, by PGRPs in *Drosophila* [41]. In this study, the insects were exposed to Cry1Ac toxin but not Bt bacteria. However, Cry1Ac alone appears to be capable of activating the Toll and IMD pathways, as the expression levels of several genes encoding PRRs and key components of these two pathways were increased by Cry1Ac exposure in the susceptible SCD strain and the two strains with lower levels of resistance to Cry1Ac (SCD-r1 and SCD-KI). Studies have shown that the insect midgut microbiota plays important roles in larvae mortality induced by Cry toxins [13,38]. The hypothesized ultimate cause of insect death is septicemia caused by midgut bacteria invading the hemocoel through toxin-induced epithelium lesions [13]. Thus, the disturbed midgut and hemolymph microbiota might be responsible for the activation of the Toll and IMD signaling pathways after Cry1Ac intoxication. Supporting this hypothesis, Cry1Ac exposure did not activate the two immune pathways in insects with high levels of resistance to Cry1Ac (C2/C3-KO). The expression of gut epithelium healing genes in the midguts of C2/C3-KO larvae was not induced by Cry1Ac exposure, indicating that the midgut cells were not damaged by the ingested toxins and, thus, the midgut bacteria could not enter the body cavity. Nevertheless, more studies are needed to clarify how Bt toxins trigger the anti-bacterial immunity in host insects.

ABC transporters are a class of transmembrane proteins that are involved in the transport of various substrates, such as amino acids, sugars, heavy metal ions and conjugates, peptides, polysaccharides, lipids and chemotherapeutic drugs across membrane [54,55]. A complete ABC transporter consists of two transmembrane domains that provide a passageway for the cargo and two cytoplasmic nucleotide-binding domains that bind and hydrolyze ATP [54]. The expression levels of 2055 genes in the midguts of the C2/3-KO larvae were significantly changed when compared to that of the SCD larvae in the absence of Cry1Ac toxins, suggesting that ABCC2 and ABCC3 play fundamental roles in the *H. armigera* midgut. In insects, ABC transporters play important roles in xenobiotic detoxification [55], which can be divided into three phases: Phase I, inactivation of xenobiotics by cytochrome P450 monooxygenases (P450s) and carboxylesterases (CESs); Phase II, conjugation of methyl, acetyl, phosphoric acid, and sulfonyl groups to xenobiotics by UDP-glycosyltransferases (UGTs) and glutathione S-transferases (GSTs); and Phase III, excretion of the metabolites through ABC transporters [56]. We found that a large number of genes related to detoxification and stress responses, including P450s, CESs, UGTs, GSTs and heat shock proteins, were constitutively overexpressed in the C2/3-KO strain relative to the SCD strain, indicating that the deletion of *HaABCC2* and *HaABCC3* might impair the excretion of metabolites from cells, thus resulting in the activation of these detoxification and stress responses.

In addition to their roles in detoxification, the insect ABC transporters are considered one of the primary receptors for several Cry toxins [57]. The ABC transporter family A2 (ABCA2) is linked to Cry2Ab resistance in *H. armigera* [58,59], and the ABC transporter family B1 (ABCB1) is associated with Cry3Aa resistance in *Chrysomela tremula* [60]. ABCC2 and/or ABCC3 are related with Cry1A resistance in several lepidopteran insects [19,20,61]. It seems that different Cry toxins use different members of the ABC transporter family of proteins as receptors. It is supposed that Cry toxin binding to ABC transporters is critical for the insertion of the “prepore” into cell membrane to form pores [15]. Supporting this hypothesis, *Bombyx mori* ABCC2 facilitated cation-permeable pore formation by Cry1A when expressed in *Xenopus* oocytes [62]. Here, we found that the number of DEGs in the midguts of the C2/3-KO larvae was much less than that of the SCD and SCD-r1 larvae after Cry1Ac exposure. Moreover, the C2/3-KO strain exhibited a strikingly different response to Cry1Ac relative to the other strains, and its midgut healing and defense pathways were not activated by Cry1Ac. It is likely that the mode of action of Cry1Ac in midguts of the C2/3-KO larvae was remarkably changed by the loss of ABCC2 and ABCC3, which might be correlated with the essential role of these two proteins in promoting pore formation by Cry toxins. These results also indicate that the binding of Cry1Ac to those ABC transporters is critical for high cytotoxicity, while interaction with cadherin may just play an enhancer role.

Tetraspanins are a family of transmembrane proteins that are implicated in cell adhesion, cell migration, signal transduction and intracellular trafficking [63,64]. They form tetraspanin-enriched microdomains by associating with each other and other membrane molecules, such as integrin, immunoglobulin superfamily proteins, signaling molecules, and other receptor proteins [65]. Structural features of tetraspanins include four transmembrane domains (TM1-TM4), one small and one large extracellular loop, and a very short intracellular loop that links TM2 and TM3 [64,65]. The large extracellular loop and TMs have been shown to be involved in tetraspanin–partner interactions and are critical for the proper function of tetraspanins [64,66]. Our RNA-seq data showed that the T92C mutation in *HaTSPAN1* changed the expression of 1254 genes in the cotton bollworm midgut, suggesting an alteration of the normal function of tetraspanin by this point mutation. The T92C mutation in *HaTSPAN1* leads to a single amino acid substitution (L31S) in TM1 of tetraspanin [22]. The TM1, TM3, and TM4 of tetraspanins typically contain polar residues that lie at internal TM interfaces, where they could form strong hydrogen bonds, thereby facilitating the proper packing of tetraspanins [65,66]. It has been shown that the interactions of TM1 with the other transmembrane domains (TM2-TM4) are required for biosynthetic maturation of tetraspanin CD82. In addition, the interactions between different TMs stabilize the conformation of the large extracellular loop [67]. It would be very interesting to investigate whether the L31S substitution in TM1 disrupts the interactions of TM1 with the other TMs, thus resulting in anomalous tertiary structure and the dysfunction of tetraspanin in *H. armigera*. Heterologous expression of the wild-type and mutant TM1 in insect cells (Sf9) and testing their interactions with the other TMs will be a good start to answer this question. In addition, a detailed comparison of the three-dimensional structures of the wild-type and mutant tetraspanins will also provide important information for addressing this issue.

Although the function of tetraspanin in cell biology and physiology has been extensively studied, the role of tetraspanin in the mode of action of and resistance to Cry toxins remains elusive. We found that many immune related genes, such as PRR and effector genes, are constitutively overexpressed in the midguts of the SCD-KI larvae. Moreover, the expression levels of those genes were strongly upregulated in the SCD-KI larvae exposed to Cry1Ac. It seems that the L31S substitution in tetraspanin confers an enhanced immune system to the SCD-KI strain. The involvement of tetraspanins in the regulation of immunity has been reported in animals, including insects. For example, the vertebrate leukocytes express about 20 tetraspanins on their surface, among which many function to regulate activation, motility and antigen presentation [68]. In insects, the *Manduca sexta* tetraspanin D76 binds to hemocyte-specific integrin and the interaction is required for cellular innate immune responses [69]. Future studies are needed to determine whether the enhanced immunity contributes to Cry1Ac resistance in the SCD-KI strain. If this is the case, then reducing the immunocompetence of resistant insects would be a promising strategy for overcoming insect resistance caused by tetraspanin mutation. A functional verification of these constitutively overexpressed PRR and effector genes by CRISPR/Cas9-mediated knockout of them in the SCD-KI strain will shed light on the role of each gene in insect resistance and provide putative targets for the development of resistance-management tactics.

## 4. Materials and Methods

### 4.1. Insect Strains and Rearing

We used four strains of *H. armigera*: three resistant strains SCD-r1, SCD-KI, C2/3-KO and a susceptible SCD strain. SCD was originally collected from Côte D’Ivoire (Ivory Coast, Africa) over 40 years ago and has been maintained in the laboratory without exposure to Bt toxins or other insecticides [27]. The SCD-r1 strain has 438-fold resistance to Cry1Ac caused by introgression of the *r1* cadherin allele into the SCD strain [27]. The SCD-KI strain was created by introduction of the T92C mutation of *HaTSPAN1* into the SCD strain using CRISPR/Cas9, and it has 125-fold resistance to Cry1Ac [22]. The C2/3-KO strain was created by knocking out both *HaABCC2* and *HaABCC3* from the SCD strain by CRISPR/Cas9, and it has >15,000-fold resistance to Cry1Ac [20]. The four *H. armigera* strains were reared in our lab (college of plant protection, Nanjing agricultural university, China). All strains were maintained as described previously [27], with larvae reared on a diet based on wheat germ and soybean powder at 27 ± 1 °C, 60 ± 10% relative humidity and a photoperiod of 16 h light: 8 h dark and adults supplied with a 10% sucrose solution.

### 4.2. Dissection of Midgut and Extraction of RNA

Larvae from different strains (SCD, SCD-r1, SCD-KI, and C2/3-KO) were starved for 8 h after molting into fifth instars. The Larvae were force-fed Cry1Ac (10 μg per insect) or PBS (negative control) and killed at 6 h after the feeding, then the midguts were dissected in 0.7% NaCl (*w*/*v*) to remove debris, transferred to 1 mL of TRIzol Reagent (Invitrogen, Carlsbad, CA, USA) and stored at −80 °C. In total, 10 midguts from 5th-instar larvae of each strain were collected as one biological replicate and each strain treated with Cry1Ac or PBS contained three biological replicates. Total RNA was extracted using the TRIzol reagent according to the manufacturer’s instructions (Invitrogen, Carlsbad, CA, USA). Subsequently, total RNA was qualified and quantified using a Nano Drop and Agilent 2100 bioanalyzer (Thermo Fisher Scientific, Waltham, MA, USA). A total of 24 samples were used for Illumina RNA-Seq and gene expression analysis.

### 4.3. mRNA Library Construction

Oligo(dT)-attached magnetic beads were used to purify mRNA. Purified mRNA was fragmented into small pieces with fragment buffer at appropriate temperature. Then first-strand cDNA was generated using random hexamer-primed reverse transcription, followed by a second-strand cDNA synthesis. Afterwards, A-tailing mix and RNA index adapters were added by incubating to end repair. The cDNA fragments obtained from previous step were amplified by PCR, and products were purified by Ampure XP beads, then dissolved in EB solution. The product was validated using Agilent 2100 bioanalyzer for quality control. The double-stranded PCR products from previous step were denatured and circularized by the splint oligo sequence to obtain the final library. The single-strand circle DNA was formatted as the final library. The final library was amplified with phi29 to make DNA nanoball (DNB) which had more than 300 copies of one molecular, DNBs were loaded into the patterned nanoarray and single end 50 base reads were generated on BGIseq500 platform (BGI-Shenzhen, China).

### 4.4. RNA-Seq Analysis

Sequencing data were filtered with SOAPnuke (v1.5.2) by: (1) removing reads containing sequencing adapter; (2) removing reads with a low-quality base ratio higher than 20%; and (3) removing reads with an unknown base ratio higher than 5%, and the remaining clean reads were obtained and stored in FASTQ format. The clean reads were mapped to the reference genome using HISAT2 (v2.2.1). Bowtie2 (v2.2.5) was applied to align the clean reads to the reference coding gene set, then the expression levels of genes were calculated using RSEM (v1.3.3) and expressed as fragments per kilobase per million (FPKM). The heatmap was drawn by R package “pheatmap” according to the gene expression in different samples. DEG analysis was performed using the R package “DESeq2” with Q value (adjusted *p* value) ≤ 0.05 and log_2_Foldchange ≥ 1. The Venn plots were drawn by R package “VennDiagram”. Functional annotation of genes was conducted with Blast2Go. KEGG (https://www.kegg.jp/, accessed on 1 August 2020) enrichment analyses of annotated DEGs were performed by R package “Phyper” based on Hypergeometric test with Q value ≤ 0.05.

## Data Availability

Short-read sequence data of RNA-seq have been deposited in the National Center for Biotechnology Information Sequence Read Archive (accession no. PRJNA826925). All other relevant data are included in the main text and supplementary materials.

## Supplementary Materials

The following supporting information can be downloaded at: https://www.mdpi.com/article/10.3390/toxins14060366/s1, Figure S1: Correlation analysis of all samples; Figure S2: Gene ontology (GO) analysis of differentially expressed genes (DEGs) between the three resistance strains and the susceptible SCD strain without Cry1Ac exposure; Figure S3: GO analysis of DEGs in Cry1Ac-treated larvae as compared to the control larvae for each strain; Table S1: Mapping statistics of mRNA libraries; Table S2: Expression levels and annotation of mapped genes; Table S3: Genes differentially expressed in the SCD-r1 strain when compared with the susceptible SCD strain; Table S4: Genes differentially expressed in the SCD-KI strain when compared with the susceptible SCD strain; Table S5: Genes differentially expressed in the C2/3-KO strain when compared with the susceptible SCD strain; Table S6: Constitutively differentially expressed genes in all three resistant strains; Table S7: Genes differentially expressed in Cry1Ac-treated SCD larvae when compared to control SCD larvae; Table S8: Genes differentially expressed in Cry1Ac-treated SCD-r1 larvae when compared to control SCD-r1 larvae; Table S9: Genes differentially expressed in Cry1Ac-treated SCD-KI larvae when compared to control SCD-KI larvae; Table S10: Genes differentially expressed in Cry1Ac-treated C2/3-KO larvae when compared to control C2/3-KO larvae; Table S11: Expression of gut epithelium healing and defense related genes.
