# Supplementary material for: Transcriptional Analysis of Cotton Bollworm Strains with Different Genetic Mechanisms of Resistance and Their Response to Bacillus thuringiensis Cry1Ac Toxin
Source: Toxins (Basel). 2022 May 25;14(6):366. doi: 10.3390/toxins14060366 (PMC9228822; doi:10.3390/toxins14060366)
Supplement: Supplementary file 1 [file toxins-14-00366-s001.zip › manuscript supplementary/Figure S1. Correlation analysis of all samples..pdf]

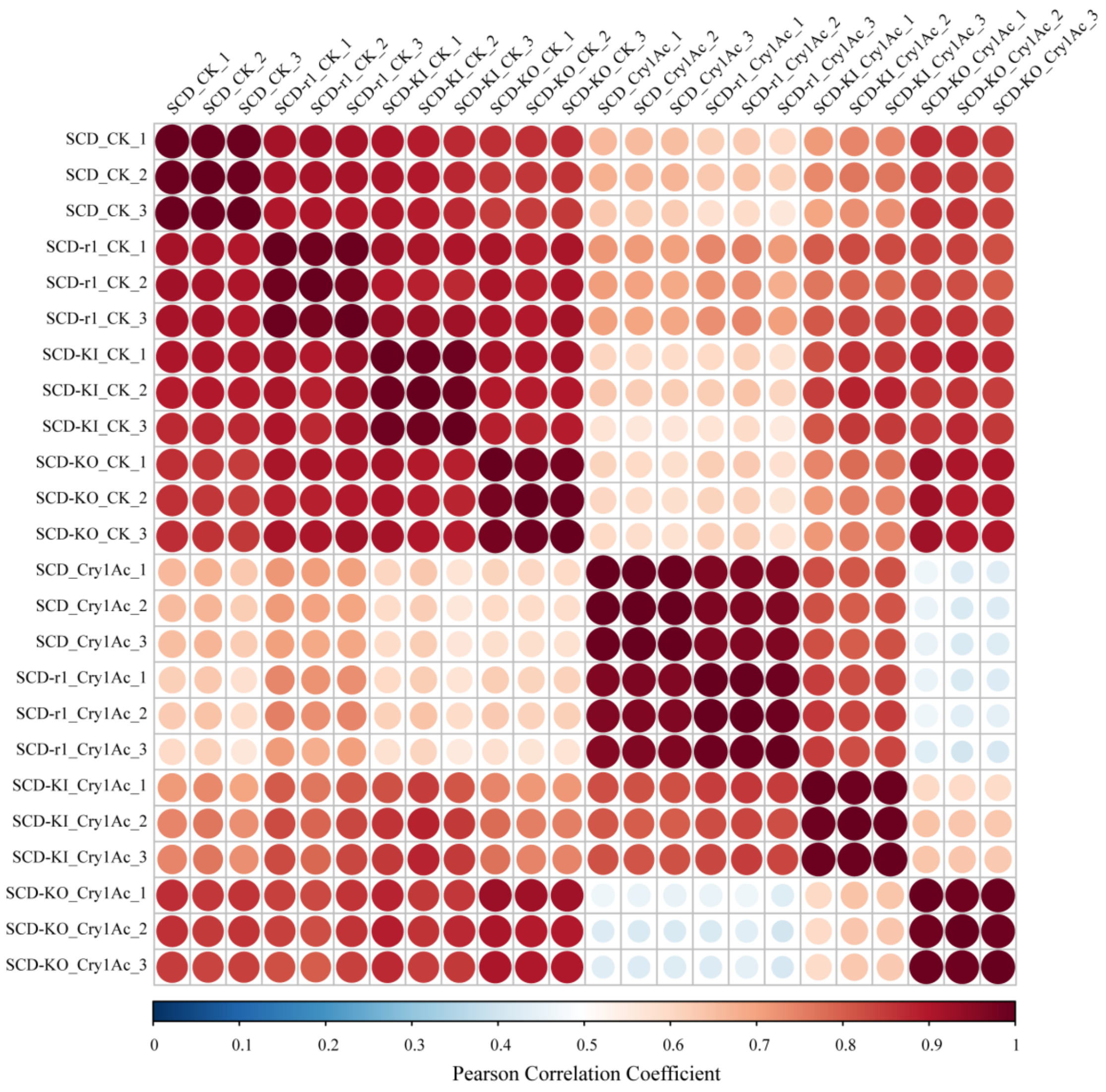

Figure S1. Correlation analysis of all samples. Coloring indicates Pearson correlation (high: red; low: blue).
