# Supplementary material for: Transcriptional Analysis of Cotton Bollworm Strains with Different Genetic Mechanisms of Resistance and Their Response to Bacillus thuringiensis Cry1Ac Toxin
Source: Toxins (Basel). 2022 May 25;14(6):366. doi: 10.3390/toxins14060366 (PMC9228822; doi:10.3390/toxins14060366)
Supplement: Supplementary file 1 [file toxins-14-00366-s001.zip › manuscript supplementary/Figure S2. Gene ontology (GO) analysis of differentially expressed genes (DEGs) between the three resistance strains and the susceptible SCD strain without Cry1Ac exposure..pdf]

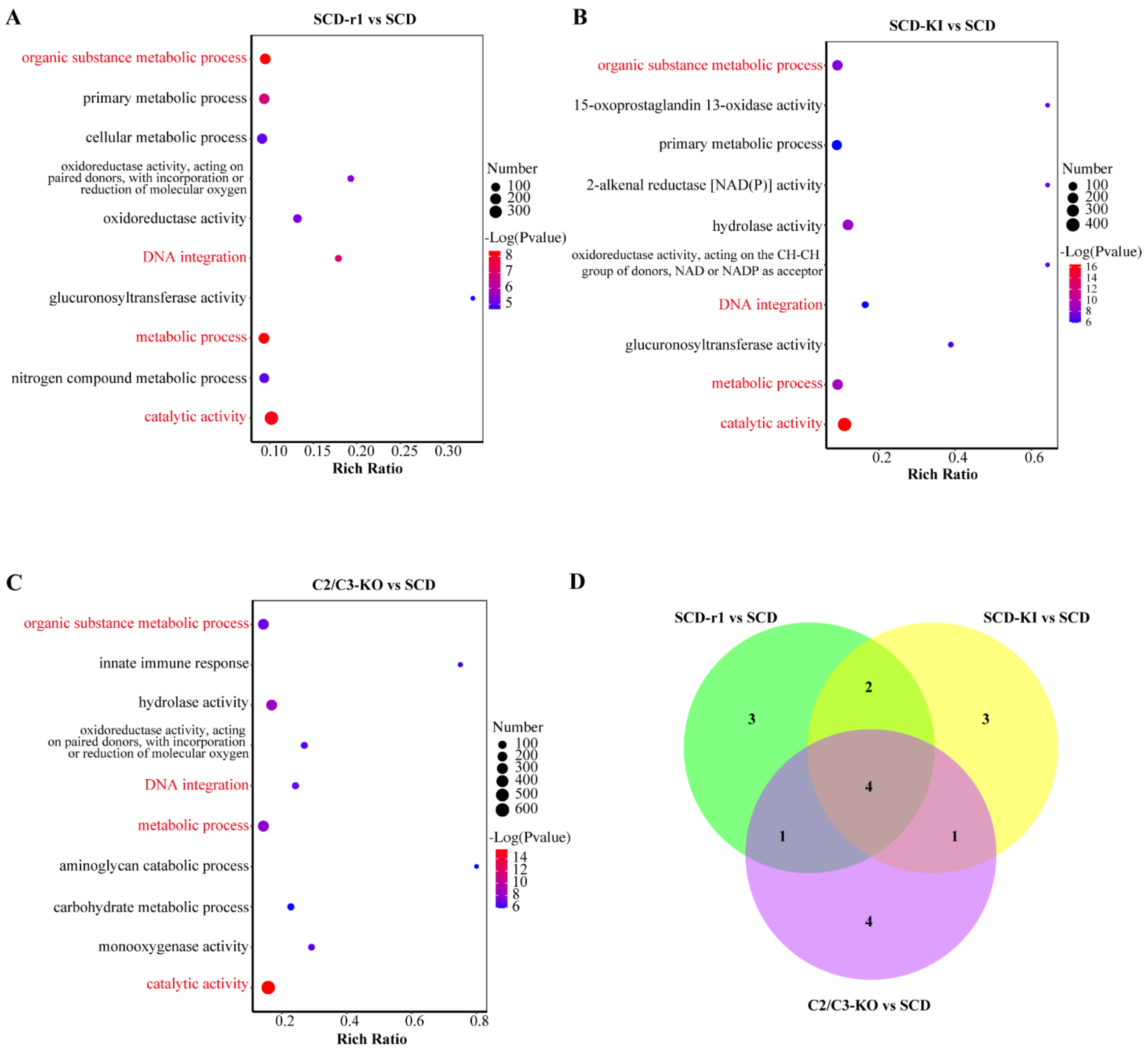

Figure S2. Gene ontology (GO) analysis of differentially expressed genes (DEGs) between the three resistance strains and the susceptible SCD strain without Cry1Ac exposure. (**A-C**) The most significantly ($p < 0.05$) enriched 10 (Top 10) GO terms for the DEGs in the three resistant strains. (**D**) Venn diagram analysis of the Top 10 GO terms enriched by the DEGs in the three resistance strains.
