# Supplementary material for: Transcriptional Analysis of Cotton Bollworm Strains with Different Genetic Mechanisms of Resistance and Their Response to Bacillus thuringiensis Cry1Ac Toxin
Source: Toxins (Basel). 2022 May 25;14(6):366. doi: 10.3390/toxins14060366 (PMC9228822; doi:10.3390/toxins14060366)
Supplement: Supplementary file 1 [file toxins-14-00366-s001.zip › manuscript supplementary/Figure S3. GO analysis of DEGs in Cry1Ac-treated larvae as compared to the control larvae for each strain..pdf]

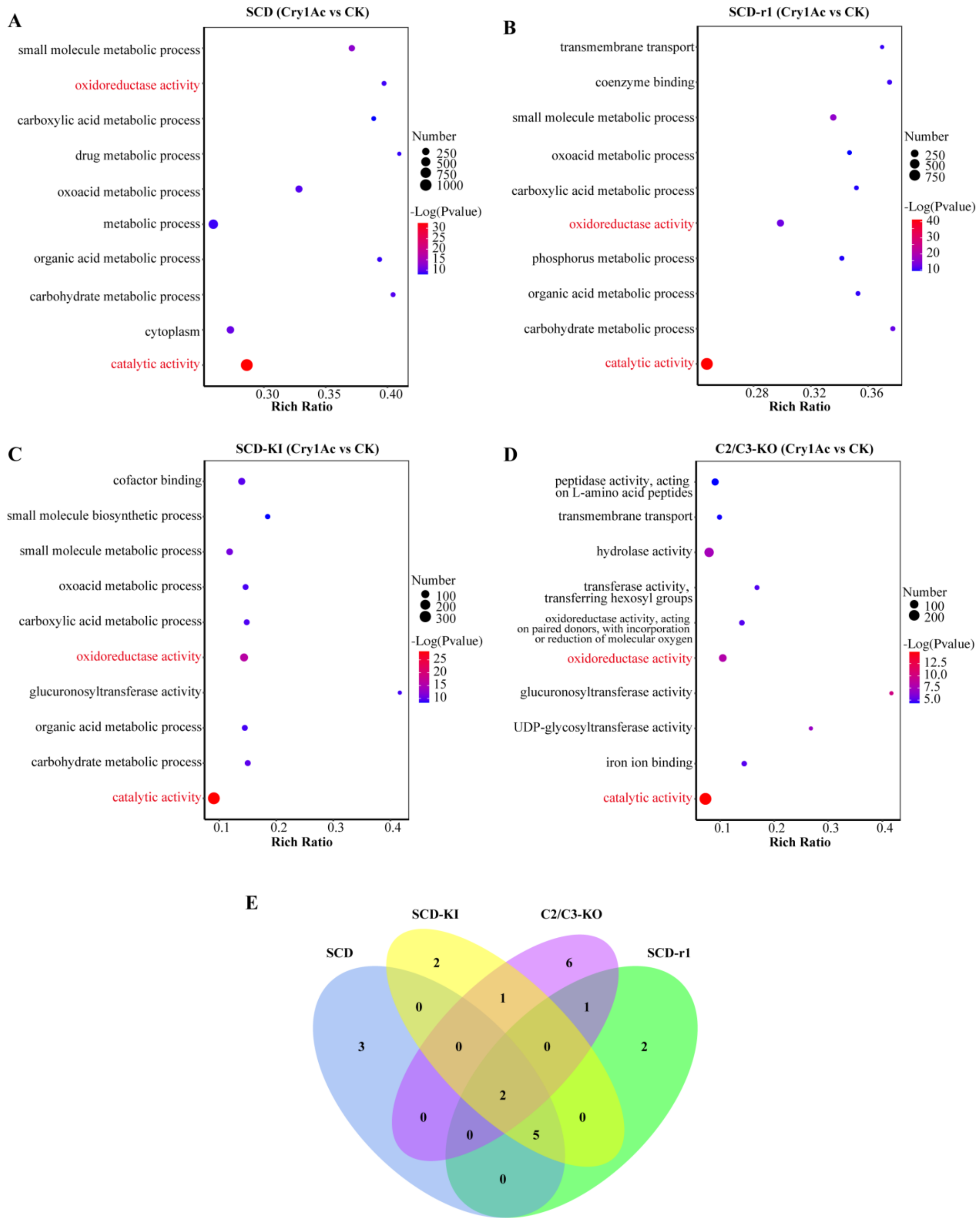

Figure S3. GO analysis of DEGs in Cry1Ac-treated larvae as compared to the control larvae for each strain. (**A-D**) The Top 10 GO terms ($p < 0.05$) for the DEGs in each strain in response to Cry1Ac exposure. (**E**) Venn diagram analysis of the Top 10 GO terms enriched by the DEGs in the four strains after Cry1Ac exposure.
